# Gene-by-Temperature Interactions and Candidate Plasticity Genes for Morphological Traits in *Drosophila melanogaster*

**DOI:** 10.1371/journal.pone.0070851

**Published:** 2013-07-30

**Authors:** Valeria Paula Carreira, Marcos A. Imberti, Julián Mensch, Juan José Fanara

**Affiliations:** 1 Departamento de Ecología, Genética y Evolución, Facultad de Ciencias Exactas y Naturales, Universidad de Buenos Aires, Instituto de Ecología, Genética y Evolución de Buenos Aires, Consejo Nacional de Investigaciones Científicas y Técnicas, Buenos Aires, Argentina; University of Lausanne, Switzerland

## Abstract

Understanding the genetic architecture of any quantitative trait requires identifying the genes involved in its expression in different environmental conditions. This goal can be achieved by mutagenesis screens in genetically tractable model organisms such as *Drosophila melanogaster*. Temperature during ontogenesis is an important environmental factor affecting development and phenotypic variation in holometabolous insects. In spite of the importance of phenotypic plasticity and genotype by environment interaction (GEI) for fitness related traits, its genetic basis has remained elusive. In this context, we analyzed five different adult morphological traits (face width, head width, thorax length, wing size and wing shape) in 42 co-isogenic single *P*-element insertional lines of *Drosophila melanogaster* raised at 17°C and 25°C. Our analyses showed that all lines differed from the control for at least one trait in males or females at either temperature. However, no line showed those differences for all traits in both sexes and temperatures simultaneously. In this sense, the most pleiotropic candidate genes were *CG34460*, *Lsd-2* and *Spn*. Our analyses also revealed extensive genetic variation for all the characters mostly indicated by strong GEIs. Further, our results indicate that GEIs were predominantly explained by changes in ranking order in all cases suggesting that a moderate number of genes are involved in the expression of each character at both temperatures. Most lines displayed a plastic response for at least one trait in either sex. In this regard, *P*-element insertions affecting plasticity of a large number of traits were associated to the candidate genes *Btk29A*, *CG43340*, *Drak* and *jim*. Further studies will help to elucidate the relevance of these genes on the morphogenesis of different body structures in natural populations of *D. melanogaster*.

## Introduction

Understanding the genetic architecture of a quantitative trait requires identifying the genes implicated in its expression in different environmental conditions [1]–[3]. This goal can be achieved by mutagenesis screens in genetically tractable model organisms such as *Drosophila melanogaster*. In fact, quantitative genetic analysis of the effects of *P*-element mutations induced in an isogenic background [4], [5] is a reliable method for functional genomic analyses [6]–[11]. In this sense, we have previously identified candidate genes related to variation of different morphological traits using 191 *P*-element insertion lines raised at 25°C [12], [13]. In general, our previous results indicate that the genetic architecture of morphological traits involves a large fraction of the genome and is largely sex- and trait-specific [12], [13].

One of the most important environmental factors affecting body size in ectothermic animals is temperature [14]. Generally, insects grown at lower temperatures are bigger [15]. The effect of temperature on different morphological traits has been profoundly studied in *Drosophila* [16]–[25]. Several of such studies were performed using *Drosophila’*s wing as a model and, while some of them showed that thermal changes affected wing shape predominantly in the posterior compartment, most of them showed that wings elongated disproportionately as temperature decreased [26]–[33].

Most of the mentioned work has been done using isofemale lines raised at different temperatures or flies derived from natural populations distributed along geographic gradients (i.e., clines). Regarding morphological traits, only a recent work has addressed the effect of genetic and environmental manipulations in *Drosophila’*s wing [34]. In particular, these authors used heterozygous insertional mutations of 16 genes involved in the formation of the wing, raising flies at two developmental temperatures [34]. Their results showed that the phenotypic effects of mutations depended on developmental temperature [34].

The phenotypic response to a change in an environmental variable (i.e., phenotypic plasticity) may vary among genotypes which may be manifested as a genotype by environment interaction (GEI) [35]–[37]. Abundant experimental and theoretical work gives strong support to the idea that GEI may be involved in the maintenance of phenotypic plasticity, genetic variation and the evolution of fitness related traits [38]–[40]. In spite of the importance of phenotypic plasticity and GEI for fitness related traits in variable environments, its genetic basis has remained elusive. In this sense, for a given trait, it is necessary to identify the genes responding to changes in different environmental variables (i.e., plasticity genes) and to establish whether they are the same genes affecting trait expression in particular conditions [1], [3], [41].

In this article, we studied different morphological traits in 42 mutants that have been previously analyzed under a different thermal treatment [12], [13] in order to investigate the genetic basis of their phenotypic plasticity. In the light of the results obtained by Debat *et al.* [34], we expected the phenotypic effects to depend on temperature. Further, and according to our previous results [12], [13], we expected this dependence to be trait- and sex-specific. Simultaneously, the experimental design employed allowed us to perform a study of GEI for each character and sex to asses if it may be involved in the maintenance of phenotypic plasticity and genetic variation. Finally, it enabled us to identify candidate genes involved in plasticity in relation to the morphological traits studied.

## Materials and Methods

### *Drosophila* Stocks

We used 42 independent homozygous viable single *p[GT1]*-element insertion lines, constructed in a coisogenic *Canton-S* background [4], to identify candidate genes affecting different morphological traits at 17°C. These lines, which are publicly available at the Bloomington *Drosophila* Stock Center (Berkeley Drosophila Genome Project website. Available: http://www.fruitfly.org. Accessed 2013 July 3), represent a random sample of the 191 lines that have been used to study the same traits at 25°C [12], [13]. All lines screened at 17°C were simultaneously assessed with one of the batches reared at 25°C. In fact, 20 out of the 42 lines were raised at both temperatures (17° and 25°C) at the same time while the remaining 22 lines were raised at 25°C within the previous six months. To account for environmental variation in morphological traits between batches, a control strain (a co-isogenic *P*-element insertion free line with the same genetic background) was run in parallel with each batch.

### Experimental Design

For each temperature, 300 pairs of sexually mature flies corresponding to each line were placed for 8 hours in separate oviposition chambers. Eggs were allowed to hatch and batches of 30 first-instar larvae were transferred to culture vials containing a standard cornmeal-agar-molasses medium (4 replicates per mutant line and 4–8 replicates for the control for each temperature). Larvae were raised at controlled temperature (4–8 replicates per line at 17±1°C and 4–8 replicates per line at 25±1°C) and 60–70% of humidity with a 12∶12 light:dark photoperiod until adult emergence. All adults emerged from each vial were preserved in a freezer at −20°C until quantification of morphological traits was performed.

### Morphological Traits

Five flies of each sex were randomly chosen from each vial (20 males and 20 females per line) and the head, the thorax and the wings of each individual were removed and placed on a slide conserving their relative position. Separate images for 3-D structures (i.e., head and thorax) and flat structures (i.e., wings) were captured using a binocular microscope (10×) and an attached digital camera connected to a computer. Different morphological traits were estimated using tpsDig [42], exactly as in previous works in which they were studied at 25°C [12], [13]. Face width (FW, the smallest distance between the eyes), head width (HW, the distance between the right and the left side of the head capsule), and thorax length (TL, the distance between the anterior margin of the thorax and the tip of the scutellum) were estimated directly from the pictures (Figure S1). For the estimation of wing size (WSi) and wing shape (WSh), 11 landmarks were digitized on the ventral face of the left wing of each fly (Figure S2). A single WSi measure (centroid size) was calculated by taking the square root of the sum of squared distances between each landmark and the centroid (the point whose coordinates are the means of the x and y coordinates of all landmarks) of each wing. Wing shape was analyzed using the Procrustes generalized least squares procedure which eliminates variation in size, position and orientation for the examination of differences in the position of landmarks [43]. This procedure generated 22 procrustes coordinates which were subsequently transformed into 18 new shape variables (relative warps, RWs) [44] using tpsRelw [45]. These variables constitute a multivariate approximation to the study of wing shape. Additionally, we estimated the Procrustes distance which represents an univariate approximation to the study of this trait.

### Statistical and Morphometrical Analyses

#### Identification of significant lines and associated candidate genes

Dunnett contrasts were performed in males and females separately, to detect significant differences (induced by *p[GT1]* insertions) between the mutants and the control in body size related traits. For WSh, one MANOVA was conducted with the RWs scores corresponding to each line and the respective control in males and females separately. Those lines that exhibited significant differences relative to the control were considered as lines bearing an insertion in a candidate gene. In order to identify these genes, we conducted homology searches of sequences flanking the *P*-element insertion against release 5 of the published *D. melanogaster* genomic sequence (http://flybase.bio.indiana.edu/). Only the gene nearest to the insertion was selected as candidate gene, except in those cases in which two genes were closer than 1 Kb to the *P*-element insertion site and neither disruption occurred in the gene.

#### Genetic correlation analyses between body size related traits

In order to include all data (estimations of different morphological traits corresponding to flies of each sex raised at 17°C and 25°C) in the same analyses, the values corresponding to each variable were transformed by subtracting from each individual value the mean value of the respective control line and dividing it by the same value.

A correlation analysis was performed between each pair of size traits within each sex and with each variable between sexes. The mean of the values corresponding to each line was used in all correlation analyses. In addition, we carried out Mantel tests to compare correlation matrices between temperatures for each sex separately using Infostat [46]. Since the diagonals of both matrices must be filled with zeros, we constructed each matrix with the respective 1 - *r* (correlation coefficient) values.

#### Visualization of wing shape deformations

Differences in wing shape between each mutant line and the respective control were estimated performing a thin-plate spline analysis using the respective Procrustes coordinates [44]. Particularly, shape changes of each line respect to the control were shown as vectors diagrams obtained with tpsSplin [47].

#### Quantitative genetic analyses

Transformed values (see above) corresponding to each univariate variable (FW, HW, TL, WSi and WSh estimated by the Procrustes distance) were analyzed using an ANOVA with line (random) and temperature (fixed) as factors in each sex separately. This procedure allowed us to estimate variance components corresponding to random sources of variation and, consequently, the percentage of total variance explained by the genetic factors (line and line by temperature interaction).

A significant GEI can arise as a consequence of differences in among-lines variance in separate environments (change in scale) and/or deviations from unity of the cross-environment genetic correlation (change in ranking order). The contribution of the two sources of variation to GEI was analyzed by means of the equation derived by Robertson [48]: *V_GEI_* = [(σ_E1_ − σ_E2_)^2^+2×σ_E1_×σ_E2_× (1 − *r_GEI_*)]/2; where *V_GEI_* is the GEI variance component, *r_GEI_* is the cross-environment genetic correlation and, σ_E1_ and σ_E2_ are the square roots of the among-lines variance components at 17°C and 25°C (which were obtained after performing ANOVAs for each temperature separately). The first term corresponds to differences in among-lines variance whereas the second to deviations from the perfect correlation across environments (*r_GEI_* <1). The cross-environment genetic correlation is the genetic correlation of measurements of the same trait in different environments and here reflects the degree in which the same genes control trait expression across temperatures. *r_GEI_* was estimated for each trait as: *r_GEI_* = COV_E1 E2_/σ_E1_ σ_E2;_ where COV_E1E2_ is the covariance of lines means for each sex measured in different temperatures.

#### Identification of candidate plasticity genes

Finally, we studied the phenotypic effect of thermal change in each mutant, sex and trait separately with a fixed ANOVA. Those lines that exhibited significant differences between temperatures were considered as lines bearing a mutation in a candidate gene involved in the plastic response of the respective character to temperature variation. A homogeneity test was conducted to compare the number of candidate lines associated to the plasticity of each trait between sexes.

In general, statistical analyses were performed using the STATISTICA software package [49]. Bonferroni correction for multiple tests was applied whenever results from multiple tests were combined in one final conclusion.

## Results

### Phenotypic Effects of Mutants at Different Temperatures and Associated Candidate Genes

**Figure 1:**
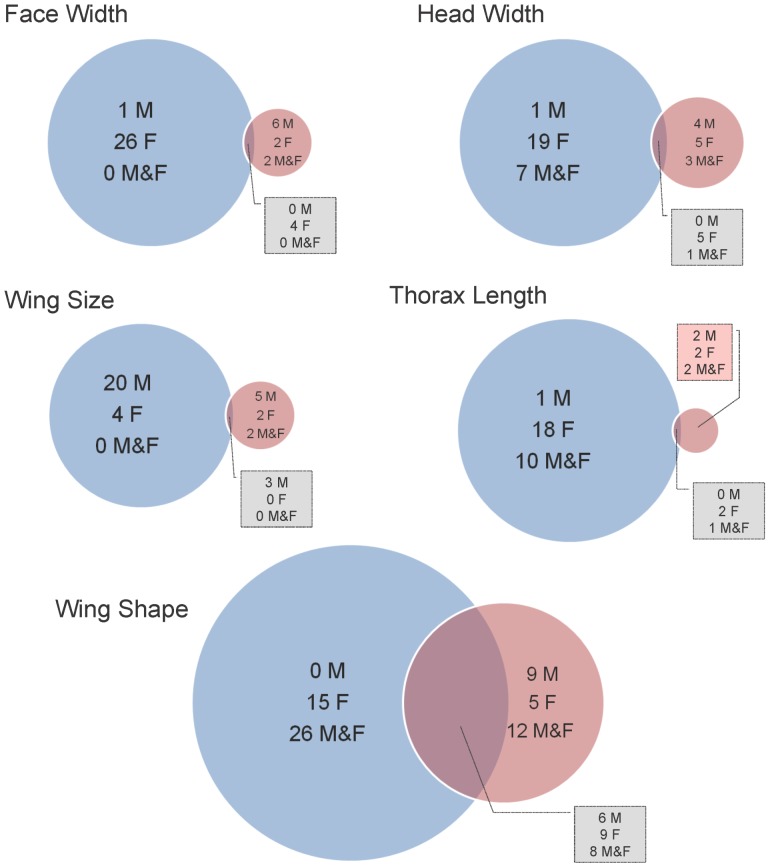
Lines showing significant effects for each trait, temperature and sex. Number of mutant lines showing significant differences with respect to the control for each morphological trait. We enumerated the lines showing significant effects at 17°C (blue circles), at 25°C (pink circles) and at both temperatures simultaneously (intersections between blue and pink circles). Also, for each one of the mentioned categories, we enumerated the lines showing significant effects only in males (M), only in females (F) and in both sexes simultanously (M&F).

The analyses revealed that all 42 lines differed from the control for at least one trait in either sex at 17°C (Table S1). Further, most of the measurements were smaller in the mutants than in the control at this developmental temperature (Table S1). Interestingly, the number of lines showing significant results at 17°C was larger than the number associated to the higher temperature (25°C, Figure 1).

We identified 46 candidate genes (39 protein coding genes) based on insertion of the *P*-element within 5 Kb from the transcription initiation site (Table S1). Nine of them (*CG13333*, *CG13334*, *CG14591*, *CG17574, clt*, *Imp*, *rut*, *SCAP* and *scyl*; Table S1) affected morphological traits only at 17°C. However, no line showed differences with respect to the control for all traits in both sexes and temperatures simultaneously. In this sense, the most pleiotropic lines were BG01011 (*Spn*), BG02462 (*CG34460*) and BG02830 (*Lsd-2*), which showed at least 13 significant differences considering all traits in both sexes and temperatures (Table S1).

As the effects of mutations on size traits were apparently different between 17°C and 25°C (Figure 1, Tables S1 and S2), we compared the genetic correlation matrices corresponding to each sex between temperatures. Results derived from Mantel tests were not significant for both sexes. These results indicate that relationships among size traits showed different patterns at 17°C and 25°C suggesting that their genetic basis differ between temperatures. Finally, results regarding WSh showed that most lines differed from the control at least in one sex and temperature (Figure 1, Table S1). However, only eight of them showed those differences in both sexes and temperatures simultaneously (Figure 1, Table S1). Even though some of these lines (BG00373, BG01014, BG02462 and BG02830) showed larger wing shape deformations than the others (BG01028, BG01354, BG01548 and BG02690), the largest changes were observed at 17°C in females (Figures S3 and S4). In general, most of the mutations displaced the posterior cross-vein (Figure S2) causing an enlargement of the distal portion of the wing at expense of the proximal part of the organ. In some cases the anterior cross-vein (Figure S2) also moved reducing the proximal region even more. Finally, some mutations produced slight veins displacements causing an anterior-posterior expansion. To conclude, it is important to note that the lines that showed larger differences in wing shape with respect to the control also exhibited significant results for other body size related traits in males and/or females grown at 17°C and/or 25°C (i.e., these mutations caused more important pleiotropic effects than the others; Table S1).

### Analyses of GEI and Identification of Candidate Plasticity Genes

In general, transformed values in flies raised at 25°C were larger than in individuals grown at 17°C (Table 1). Considering the transformation implemented, these results indicate that the mutant lines grew less than the control at 17°C while the opposite occurred at 25°C. The line factor was significant in a few cases but the line by temperature interaction was highly significant for all traits in both sexes implying a significant contribution of the genetic factors to total phenotypic variation (Table 1).

**Table 1:** Principal results of the ANOVAs for morphological traits at 17°C and 25°C in each sex separately.

	FaceWidth	HeadWidth	ThoraxLength	WingSize	WingShape
Females					
**L**	1.25	1.36	1.15	1.82* (10)§	1.49
**T**	63.72***	17.99***	40.54***	1.07	159.97***
**L×T**	4.40*** (18)	5.24*** (21)	6.86*** (27)	4.88*** (18)	3.13*** (12)
Males					
**L**	1.56	1.94* (12)	2.56** (14)	1.71* (11)§	2.88*** (12)
**T**	11.70**	4.13* §	42.28***	30.62*** ^†^	114.78***
**L×T**	5.03*** (19)	6.09*** (22)	3.92*** (14)	7.65*** (27)	2.38*** (7)

The *F* value and its significance as well as the percentage of total phenotypic variance explained by each random source of variation (between parentheses) are shown. L: Line, T: Temperature.

* p<0.05,

** p<0.01,

*** p<0.001.

§ Not significant after Bonferroni correction for multiple tests (P_B_ = 0.025). The mean of the transformed values at 25°C was larger than the mean at 17°C in all cases except for †.

**Figure 2:**
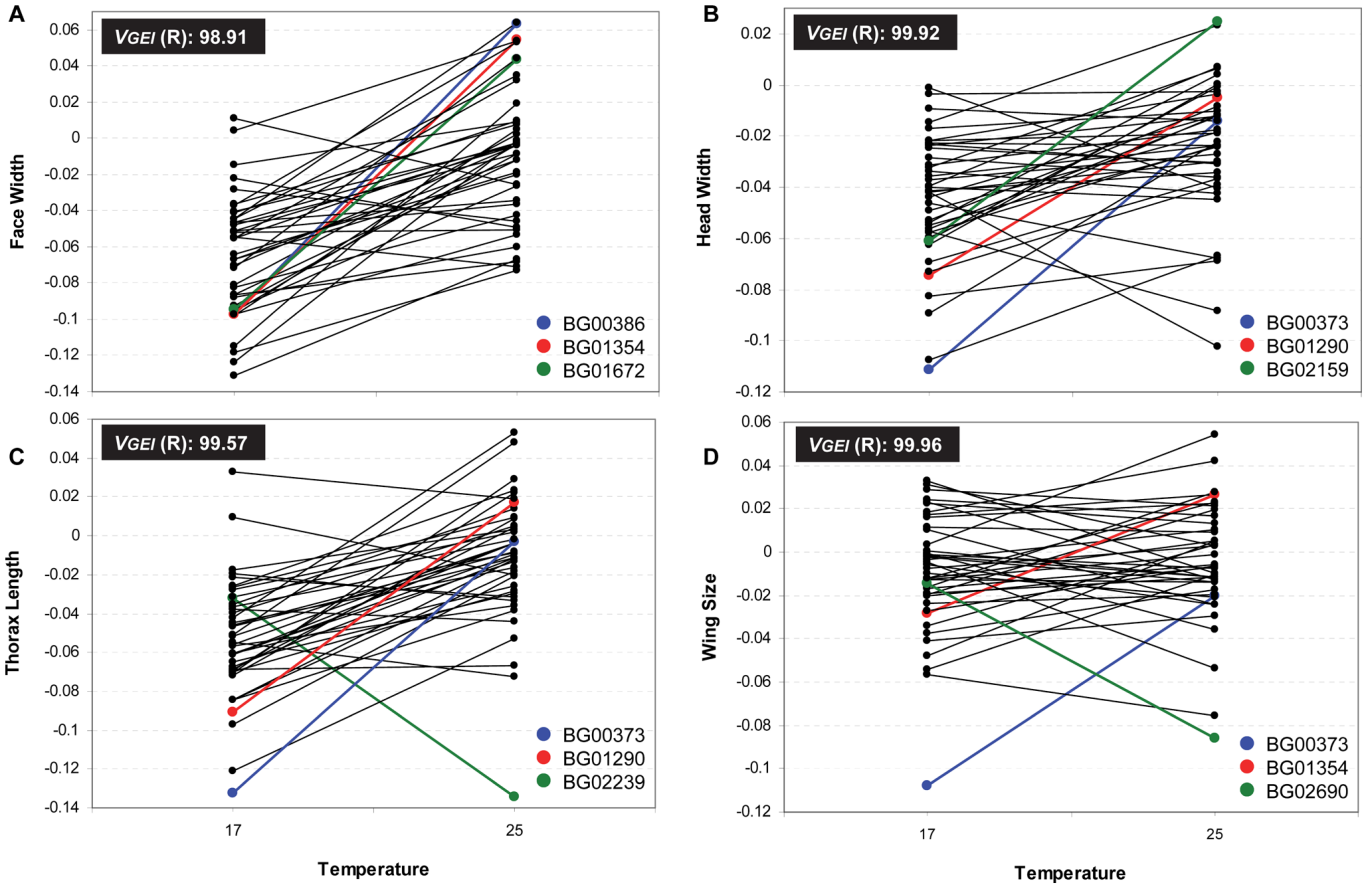
Genotype by environment interaction for each body size related trait in females. Line by temperature interaction in females for A) Face Width, B) Head Width, C) Thorax Length and D) Wing Size. Each dot corresponds to the average of the transformed values. *V_GEI_* (R) is the percentage of GEI’s variance explained by changes in ranking order. The three lines showing the largest significant differences between temperatures are coloured.

**Figure 3:**
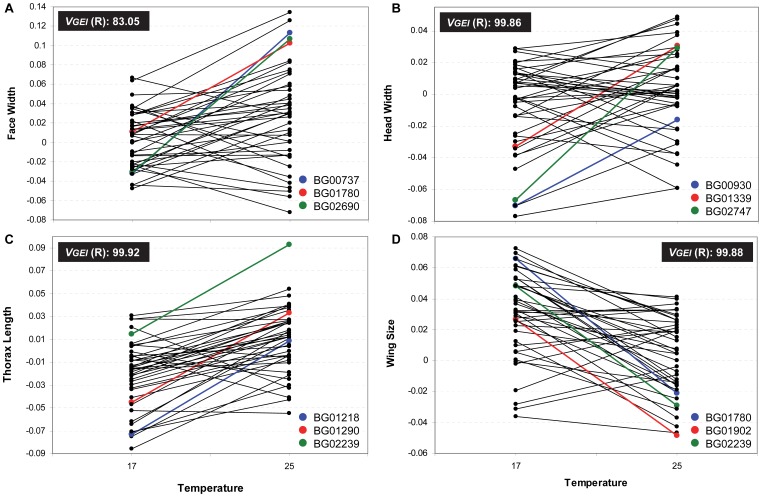
Genotype by environment interaction for each body size related trait in males. Line by temperature interaction in males for A) Face Width, B) Head Width, C) Thorax Length and D) Wing Size. Each dot corresponds to the average of the transformed values. *V_GEI_* (R) is the percentage of GEI’s variance explained by changes in ranking order. The three lines showing the largest significant differences between temperatures are coloured.

**Figure 4:**
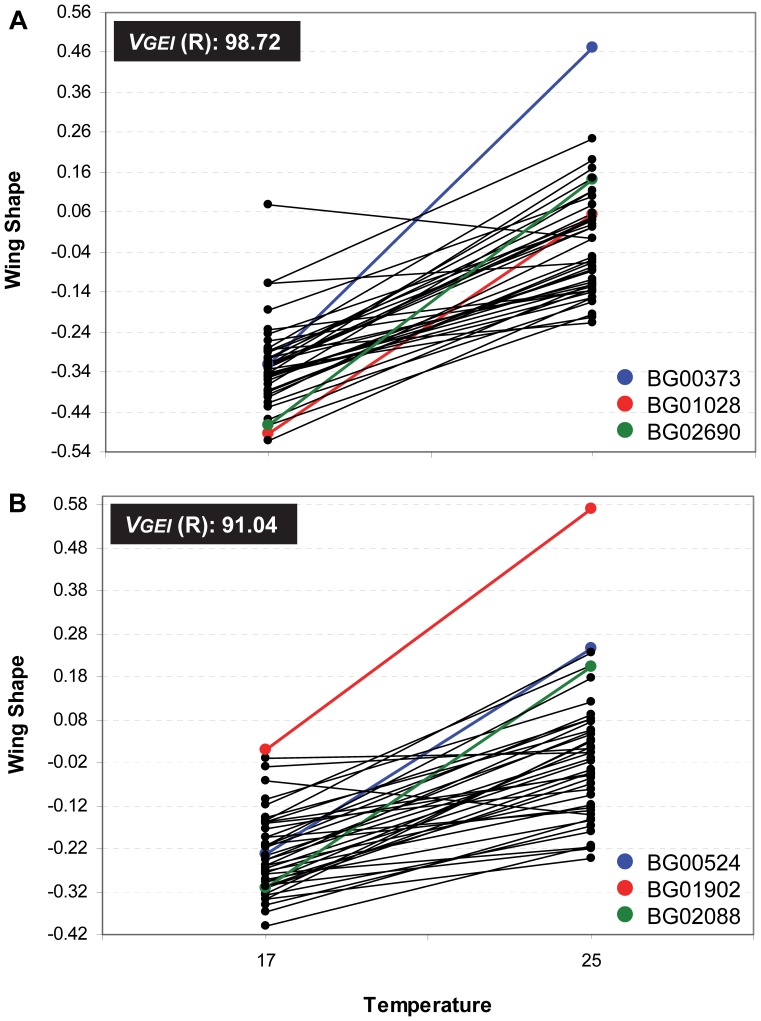
Genotype by environment interaction for wing shape in both sexes. Line by temperature interaction for wing shape (estimated by the procrustes distance) in A) females and B) males. Each dot corresponds to the average of the transformed values. *V_GEI_* (R) is the percentage of GEI’s variance explained by changes in ranking order. The three lines showing the largest significant differences between temperatures are coloured.

We observed low genetic correlations (*r_GxE_*) between measurements of each trait in different environments (17°C and 25°C; Table S3). Further, the change in ranking order (i.e., the deviation from perfect correlation between temperatures) explained a percentage of GEI’s variation much larger than the change in scale (i.e., the difference in variance among lines between temperatures; Table S3). This pattern may be easily seen in Figures 2, 3 and 4 which show the mean of the transformed values corresponding to each line at both temperatures for every morphological trait in females and males.

We identified 39 lines displaying a plastic response for at least one morphological trait in either sex (Table S4). According to the homogeneity test, the number of lines showing plasticity for each trait differed between sexes (χ^2^_4_ = 11.5, p = 0.02) suggesting that the genetic basis underlying plasticity for these characters is sexually dimorphic.

**Figure 5:**
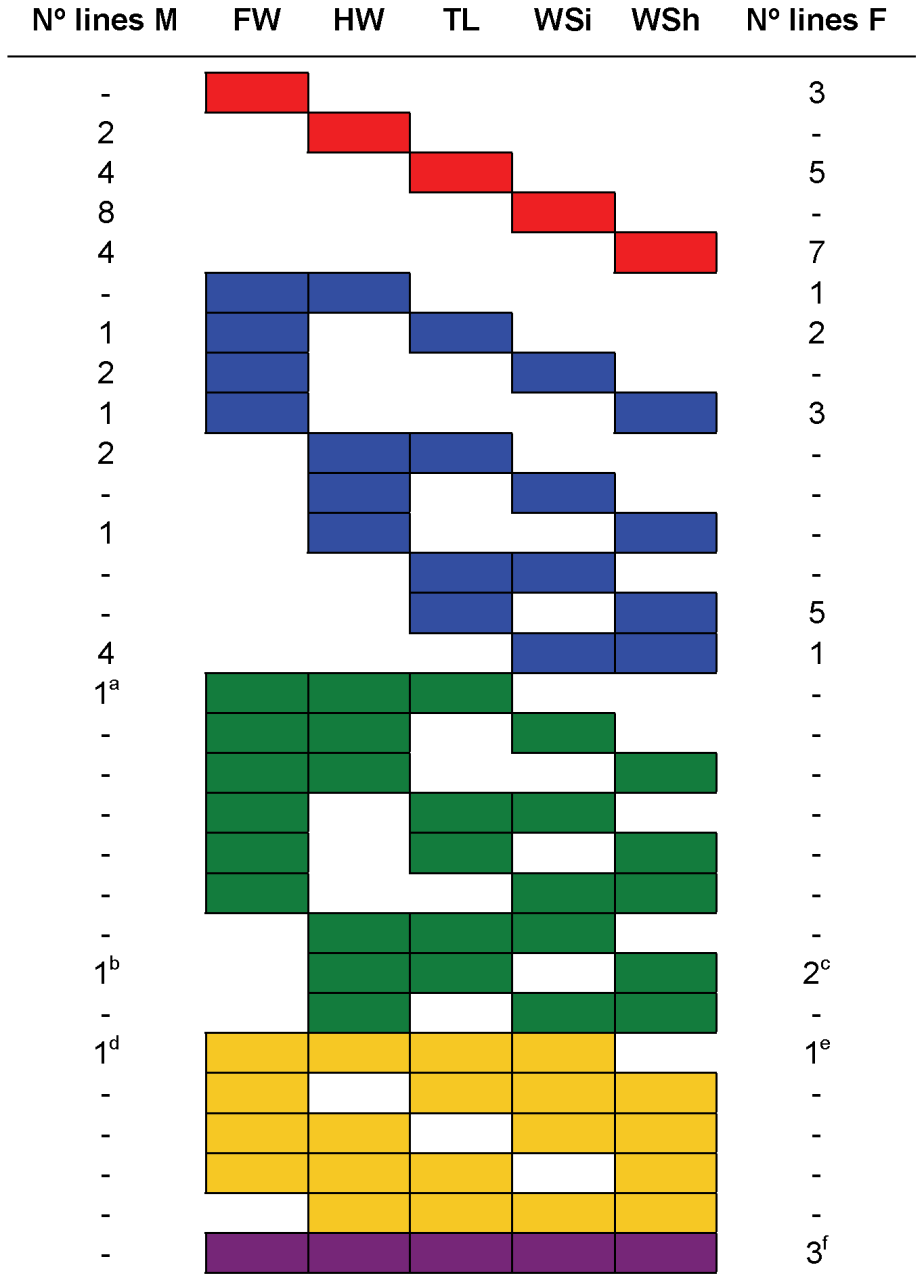
Lines showing phenotypic plasticity for different traits in each sex. Number of lines showing phenotypic plasticity for one, two, three, four or all five traits in females (F) and males (M). FW: Face Width, HW: Head Width, TL: Thorax Length, WSi: Wing Size and WSh: Wing Shape. The candidate genes (or the name of the lines when the respective genes could not be identified) affecting plasticity of a large number of traits in each sex are: ^a^ *Drak*; ^b^ *rut*; ^c^ BG00930, *CG13333*, *CG13334*; ^d^ BG02239; ^e^ *Btk29A*; ^f^ *CG43340*, *Drak*, *jim*.

The line that showed a plastic response for more morphological traits in both sexes is BG02159 (Figure 5, Table S4), in which the *P*-element insertion ocurred in *Drak* (Table S1). In females, BG00373 (*jim*) as well as BG01354 (*CG43340*) displayed a plastic response for all five morphological traits whereas BG01290 (*Btk29A*) showed differences between temperatures in four of them (Figure 5, Table S4). In contrast, males did not present any line displaying a plastic response for all traits although BG02239 showed differences between thermal treatments in four of them (Figure 5, Table S4). It is important to note that these lines are among those that showed the largest significant differences between temperatures (Figures 2, 3 and 4; Table S4).

## Discussion

### Genetic Basis of Morphological Traits at Different Temperatures

Our results suggest that many genes are related to variation of morphological traits at 17°C and that their effect depends extensively on the sex, according to observations made at 25°C [12], [13]. However, analyses comparing results obtained at different temperatures indicate that the relationships among body size related traits as well as their genetic basis differ between 17°C and 25°C. These results suggest that relations among growth rates of different imaginal discs may change with temperature of development in flies. Therefore, our results are in line with those obtained by Shingleton *et al.* [50], who have found that different environmental factors, including temperature, might regulate body and trait size as well as the relationship between them (i.e., allometry) through different developmental mechanisms. Further, our results indicate that the genetic basis involved in this process might differ between distinct environmental states (i.e., 17°C and 25°C).

Regarding wing shape, our results are in agreement with previous studies [51], [52] in that most mutations analyzed determined small but significant phenotypic effects and that morphometric variation show, simultaneously, a high degree of integration across the wing as well as certain local specificity [53]–[59]. In particular, each mutation might affect wing shape more globally or locally depending on the position of the gene in the genetic hierarchy that determine the development of the organ and the capacity of the system to buffer the effects in different instances of that process [13]. In this sense, previous studies suggest that developmental buffering is a trait-specific process [60], [61] that may be altered by environmental as well as genetic factors [34]. Finally, there seems to be a great resemblance between our results and those obtained by Debat *et al.* [34]. Furthermore, other authors have found similar wing shape changes when studying *Drosophila* populations located along different latitudinal gradients [26], [57], [62]–[63]. Therefore, these observations suggest that the same pathways may be involved in phenotypic variation observed in nature and in experimental populations, as it was observed for developmental time [64].

### Candidate Genes for Plasticity of Morphological Traits

The analyses revealed that the control line tended to grow significantly more than the mutant lines at 17°C, while the control flies reared at 25°C showed sizes lying in the middle of the range of genotypic effects. This is interesting, especially because the control line was raised together with 20 lines at both temperatures at the same time, giving support to the idea that the insertions effect could be responsible for the plastic response. However, it should be noticed that the control line has been kept for 10 years as an isogenic stock and, during this period of time, it might have accumulated novel mutations affecting its thermal plastic response. In spite of this, our observations are remarkably similar to those made by Debat *et al.* using a different set of mutant lines raised at 18°C and 28°C [34]. Beyond this strange pattern, flies were generally larger at lower temperatures, according to multiple findings [16], [17], [19], [21], [25]. Particularly, size increment showed by the control line was comparable to that observed in isofemales lines of *D. melanogaster* studied in the mentioned works. Finally, as WSh analysis presented analogous results, we stress that both, the traits and the effects of the mutations, exhibited differences between temperatures (i.e., plasticity was observed at two levels of analysis).

Analyses of GEI showed that the genetic correlation between the values of each trait measured at different temperatures was relatively low indicating that a moderate number of genes are associated to variation of each character at both temperatures. The type of GEI observed indicate that, if natural populations present analogous genetic variants, selection might favor different genotypes in environments with distinct temperatures contributing to maintain intra-specific genetic variability [38], [65]. Finally, this might help to explain the clinal patterns repeatedly observed in *Drosophila* for different traits, including the morphological ones [66]–[75]. This would occur through the process of genetic assimilation [76] which seems to be an appealing mechanism for body size evolution based on abundant evidence of GEIs for traits in general and body size in particular [77].

Results of the line-specific analyses suggest that most genes involved in the genetic basis of morphological traits cause disparate phenotypic effects at different temperatures (i.e., plastic effects) although these effects are highly trait- and sex-specific. This seems to contradict results derived from a recent study of variation in genome-wide gene expression of an outbred *D. melanogaster* population under 20 different environments [78] which revealed that only ∼15% of the transcriptome is environmentally plastic. However, discrepancies might be explained by differences in the experimental designs implemented (i.e., plasticity might be due to differences in post-translational modifications between environments instead of transcriptional differences which can not be assesed by the methodology implemented in the mentioned work). Furthermore, the mentioned work analyzed the transcriptome in adults, whereas the developmental stages relevant for our study are the larval and pupal stages, when the growth of imaginal discs occurs. Setting these differences aside, the cited work grouped transcripts showing phenotipic plasticity into two categories: Class I, in which transcripts are genetically variable and Class II, in which transcripts have low genetic variance and show sexually dimorphic expression [78]. In particular, only four of our candidate genes showed significant results in the mentioned investigation: on one side *CG13333*, which was assigned to Class II in males and, on the other side, *Hsp 27*, *CG17574* and *Lsd-2*, which were nominated as Class I genes in both sexes [78]. Furthermore, concerning these genes, *Lsd-2* was the only one which was associated with developmental time and starvation resistance [78]. This is interesting because we have found that this gene displayed highly pleiotropic effects on morphological traits closely associated to body size, a character also related to the mentioned life history traits [79]–[84]. It is worth pointing out that this gene did not show outstanding results with respect to phenotypic plasticity, a pattern which was also shown by *Spn* and *CG34460*, the other most pleiotropic genes for morphological traits. These observations are somewhat in line with preliminary results that showed that *Lsd-2* present low nucleotidic polymorphism as well as little genetic differentiation among natural populations of *D. melanogaster* (Carreira, unpublished data). Furthermore, an exploratory analysis of the sequence of this gene in different *Drosophila* species indicates that its evolution has occurred according to the postulates of the neutral theory (Carreira, unpublished data) which is in agreement with recent works that did not find evidence of positive selection for *Lsd-2* in *D. melanogaster* [41] and related species [85].

On the contrary, the few genes that affected plasticity of a large number of traits did not affect many characters in each temperature. This is the case of *Btk29A*, *CG43340* and *jim* in females; the unidentified gene affected by the *P*-element insertion in BG02239 in males and *Drak* in both sexes. It would be interesting to investigate if these genes also present important nucleotidic variability in natural populations and thus are less conserved than the others. However, some genes might present high levels of genetic variability for a trait and, concurrently, low levels of plasticity for it which might indicate that the character is subjected to environmental canalization. Studies on the molecular genetics of populations of these candidate genes might help to clarify this and other issues regarding evolution of morphological traits.

## Supporting Information

Figure S1 **Head and thorax of a fly and related morphological traits.** Picture showing the positioning of 3-D body structures on a slide and related measurements taken with tpsDig.
(TIF)

Figure S2 **Ventral view of left wing and positioning of landmarks.** LV: longitudinal vein, HCV: humeral cross vein, ACV: anterior cross-vein, PCV: posterior cross-vein.
(TIF)

Figure S3 **Lines showing wing shape deformations at both temperatures in females.** Lines showing significant wing shape deformations with respect to the control line in females raised at 17°C (in blue) and 25°C (in red). The gene affected by the *P*-element insertion is shown between parentheses for each line. Arrows indicate the magnitude and direction of landmarks displacement with respect to the corresponding control line. Arrows size has been magnified three times to show more clearly wing shape changes.
(TIF)

Figure S4 **Lines showing wing shape deformations at both temperatures in males.** Lines showing significant wing shape deformations with respect to the control line in males raised at 17°C (in blue) and 25°C (in red). The gene affected by the *P*-element insertion is shown between parentheses for each line. Arrows indicate the magnitude and direction of landmarks displacement with respect to the corresponding control line. Arrows size has been magnified three times to show more clearly wing shape changes.
(TIF)

Table S1 **Lines in which the** ***P*** **-element insertion affected one or more morphological traits in either sex at 17°C.** The candidate gene, the site of the mutation and its morphological effect are given for each line.
(PDF)

Table S2 **Principal results of genetic correlation analyses between body size related traits at 17°C and 25°C.** Correlation coefficients corresponding to the analyses within each sex and between sexes for each variable are shown.
(PDF)

Table S3 **Principal results of ANOVAs for morphological traits in each temperature and sex separately and analyses of GEI.** The *F* values and the genetic variance components derived from the ANOVAs are shown. Also, the correlation coefficients and the components explaining the interaction between temperatures are given.
(PDF)

Table S4 **Principal results of the ANOVAs performed to study the change of the phenotypic effect of the** ***P*** **-element insertion with thermal change in each line, sex and trait separately.** The unsigned difference between the means of the transformed values at 17°C and 25°C for each morphological trait are given.
(PDF)
