# Supplementary figures and images for: Gene-by-Temperature Interactions and Candidate Plasticity Genes for Morphological Traits in Drosophila melanogaster
Source: PLoS One. 2013 Jul 30;8(7):e70851. doi: 10.1371/journal.pone.0070851 (PMC3728209; doi:10.1371/journal.pone.0070851)

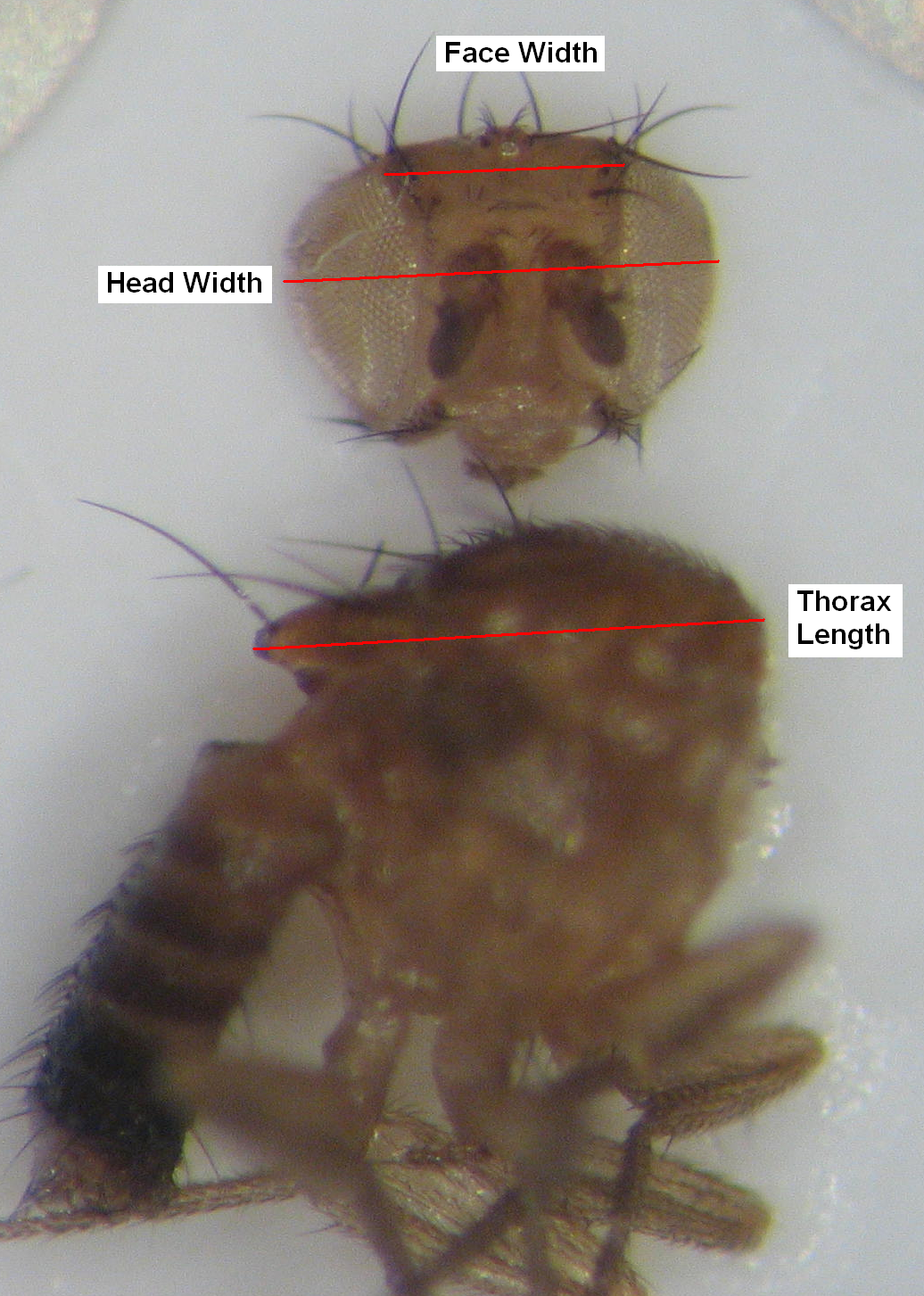

Supplement: Figure S1 — Head and thorax of a fly and related morphological traits. Picture showing the positioning of 3-D body structures on a slide and related measurements taken with tpsDig. (TIF) [file pone.0070851.s001.tif]

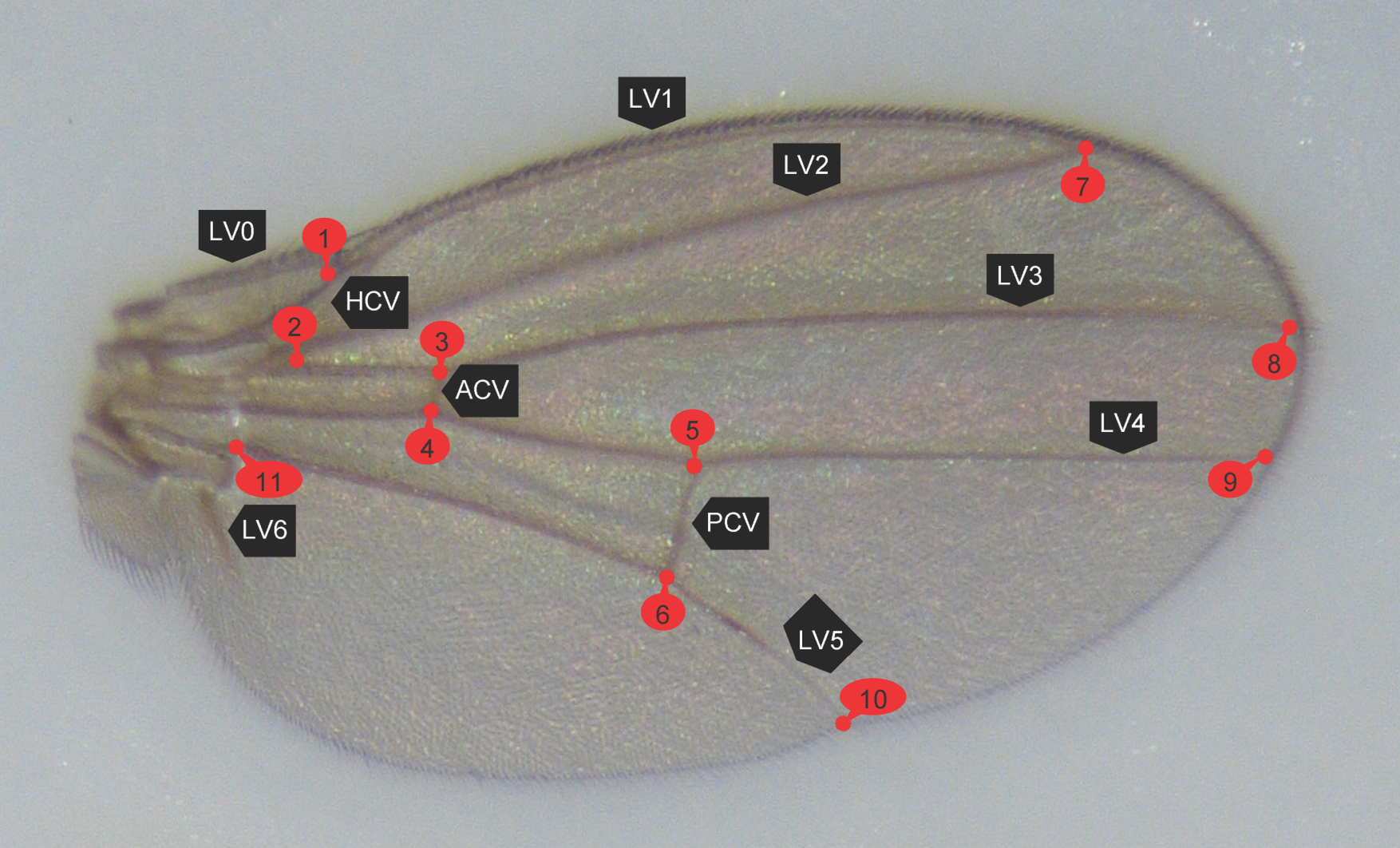

Supplement: Figure S2 — Ventral view of left wing and positioning of landmarks. LV: longitudinal vein, HCV: humeral cross vein, ACV: anterior cross-vein, PCV: posterior cross-vein. (TIF) [file pone.0070851.s002.tif]

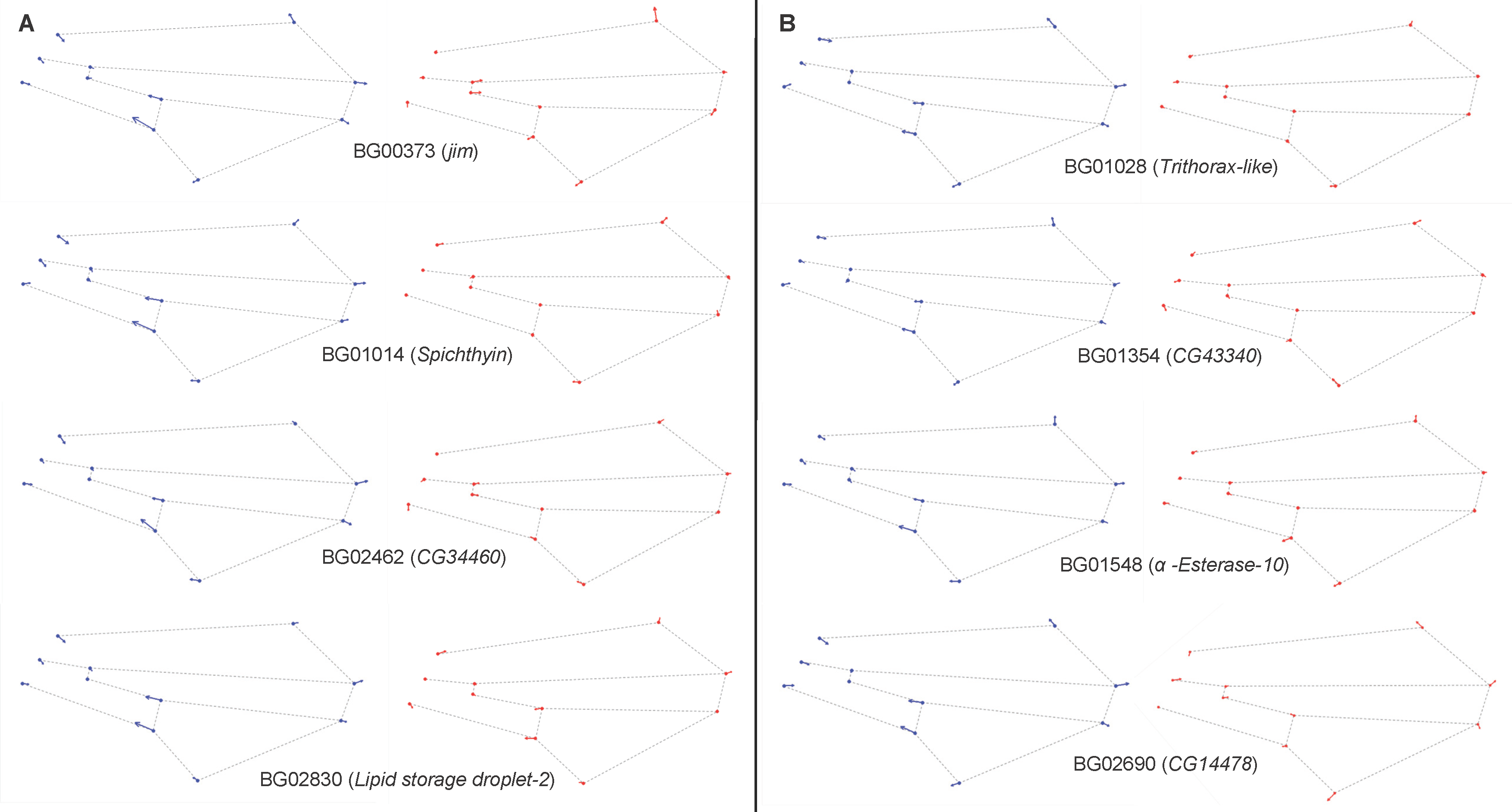

Supplement: Figure S3 — Lines showing wing shape deformations at both temperatures in females. Lines showing significant wing shape deformations with respect to the control line in females raised at 17°C (in blue) and 25°C (in red). The gene affected by the P-element insertion is shown between parentheses for each line. Arrows indicate the magnitude and direction of landmarks displacement with respect to the corresponding control line. Arrows size has been magnified three times to show more clearly wing shape changes. (TIF) [file pone.0070851.s003.tif]

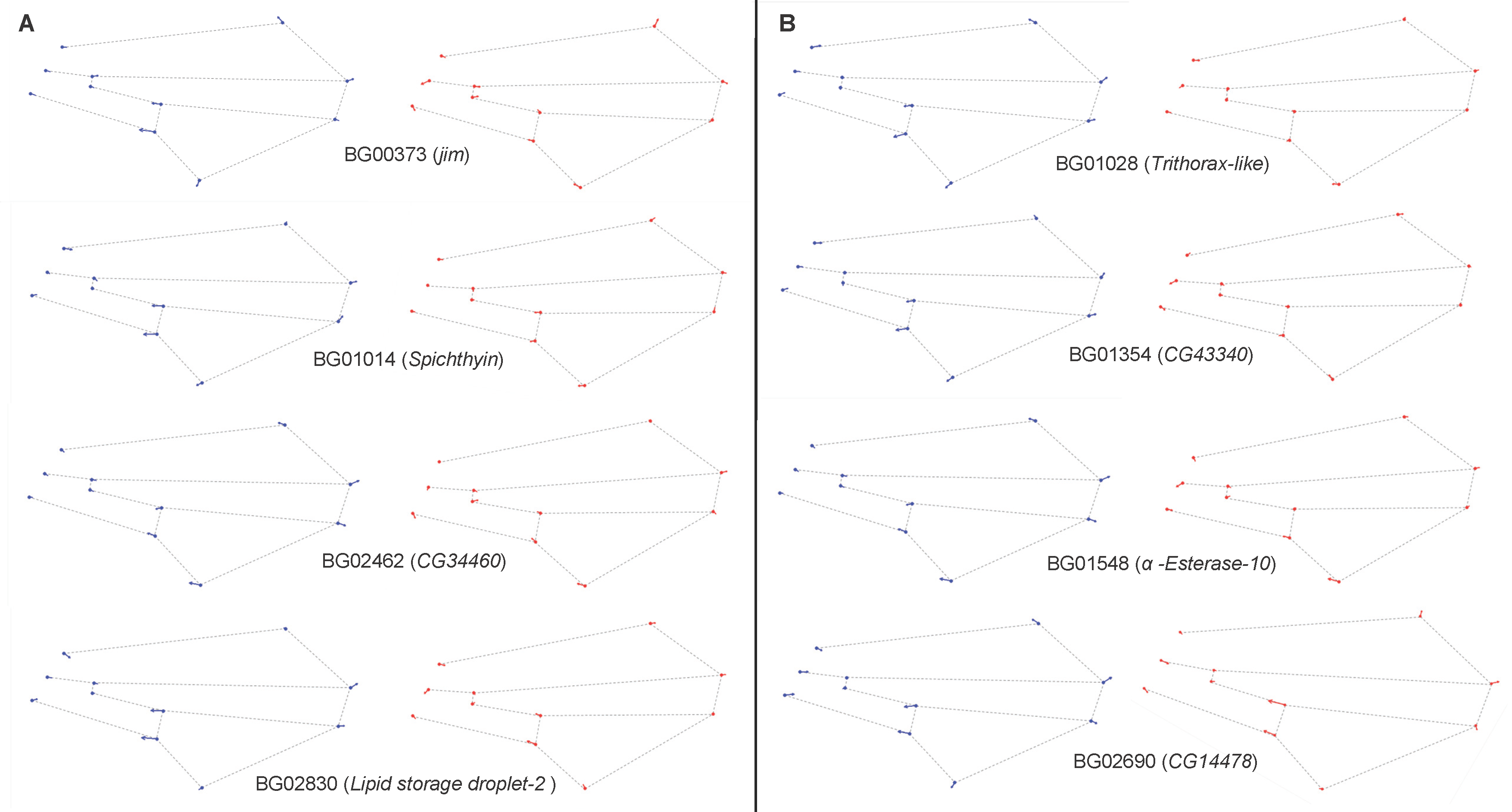

Supplement: Figure S4 — Lines showing wing shape deformations at both temperatures in males. Lines showing significant wing shape deformations with respect to the control line in males raised at 17°C (in blue) and 25°C (in red). The gene affected by the P-element insertion is shown between parentheses for each line. Arrows indicate the magnitude and direction of landmarks displacement with respect to the corresponding control line. Arrows size has been magnified three times to show more clearly wing shape changes. (TIF) [file pone.0070851.s004.tif]
