# Supplementary material for: Gene-by-Temperature Interactions and Candidate Plasticity Genes for Morphological Traits in Drosophila melanogaster
Source: PLoS One. 2013 Jul 30;8(7):e70851. doi: 10.1371/journal.pone.0070851 (PMC3728209; doi:10.1371/journal.pone.0070851)
Supplement: Table S1 — Lines in which the P -element insertion affected one or more morphological traits in either sex at 17°C. The candidate gene, the site of the mutation and its morphological effect are given for each line. (PDF) [file pone.0070851.s005.pdf]

Table S1. Lines in which the *P*-element insertion affected one or more morphological traits in either sex at 17°C.

Line	Candidate Gene	*p[GT1]* insertion	Cytogenetic map	Face Width		Head Width		Thorax Length		Wing Size		Wing Shape	
				♂	♀	♂	♀	♂	♀	♂	♀	♂	♀
BG00369	*CG13333 / CG13334*	671 bp at 3' side / 532 bp at 5' side	50B1	ns	▼	ns	▼	ns	▼	▲	ns	S	S
BG00373	*jim*	303 bp at 5' side	80A4	ns	▼	▼	▼	▼	▼	ns	▼	S*	S*
BG00386	*NMDA receptor 1* (*Nmdar1*)	348 bp at 3' side	83A6-A7	ns	▼	ns	▼	ns	▼	ns*	▼	S*	S
BG00524	*tal-1A*	647 bp at 5' side	87F14	ns	ns	ns	ns	ns	▼	▲	ns	ns*	S*
BG00737	*Heat shock protein 27* (*Hsp 27*)	127 bp at 5' side	67B3	ns*	▼	▼	▼	▼	▼	ns	ns	S*	S
BG00930	No sequence			ns	▼	▼	▼	▼	▼	ns	▼	ns	S*
BG00992	*CG17574*	In gene (intron)	49D4-49D6	ns	ns	ns	ns	ns	ns	▲	ns	ns	S
BG01011	*Spinophilin* (*Spn*)	33 bp at 5' side	62E6-E7	ns	▼*	▼	▼*	▼	▼*	ns*	ns*	ns*	S*
BG01014	*Spichthyin* (*spict*)	In gene (exon)	33F3	▲	ns	ns	ns	ns	ns	▲	ns	S*	S*
BG01028	*Trithorax-like* (*Trl*)	In gene (intron)	70F4	ns	▼	ns	▼	ns	▼	▲	ns	S*	S*
BG01081	*Glutamate oxaloacetate transaminase 1* (*Got1*)	In gene (exon-intron)	52E7-E10	ns	▼	ns	▼	ns	▼	ns	ns	S*	S
BG01214	*sugarless* (*sgl*)	In gene (5´UTR)	65D4-D5	ns	▼	ns	▼	▼	▼	ns	ns	S*	S
BG01218	*CG6767*	In gene (intron)	67C4-C5	ns	▼	▼	▼	▼	▼	ns	ns	ns*	S*
BG01290	*Btk family kinase at 29A* (*Btk29A*)	In gene (intron)	29A1-A3	ns	▼	ns	▼	▼	▼	ns	▼	ns*	S
BG01339	*cricklet* (*clt*)	In gene (5´UTR)	57F4	ns	▼	ns	▼	ns	▼	ns	ns	S	S
BG01354	*CG43340*	694 bp at 5´ side	43E6-E7	ns	▼	ns	▼	ns	▼	▲	ns	S*	S*
BG01488	No similarity found			ns	▼	ns	▼	ns	▼	ns	ns	S	S

(Continued)

Table 1 continued

BG01548	*α -Esterase-10* (*α-Est10*)	In gene (intron)	84D8-D9	ns	ns	ns	ns*	ns	▼	▲	ns	S*	S*
BG01573	*forkhead box, sub-group O* (*foxo*)	6418 bp at 5’ side	88A5-A8	ns	▼	▼	ns	▼	▼*	ns	ns*	ns	S
BG01672	*SCAP / CG14591*	113 bp at 5’ side / 28 bp at 3’ side	42A8	ns	▼	ns	▼	ns	ns	▲	ns	S	S
BG01683	*CG32572*	In gene (intron)	15A3	ns	ns	ns	ns	ns	▼	▲	ns	S*	S
BG01716	*PAPS synthetase* (*Papss*)	780 bp at 3’ side	76D1-D2	ns	ns	ns	ns	ns	ns	▲	ns	ns*	S*
BG01726	*CG11382*	197 bp at 5’ side	1E4	ns	ns	ns*	ns	ns*	ns	▲*	ns	S	S*
BG01735	*CG13130 / big brain* (*bib*)	In genes (5´UTR)	30F5	ns*	ns	ns*	ns	ns*	ns	▲	ns	S	S
BG01780	*CG11226*	In gene (exon)	80A4	ns*	ns	ns	ns	ns	ns	▲	ns	S	S
BG01822	*IGF-II mRNA-binding protein* (*Imp*)	In gene (intron)	9F1-9F4	ns	▼	ns	▼	ns	▼	ns	ns	S	S
BG01902	*Mastermind* (*mam*)	In gene (intron)	50C23-D3	ns	ns	ns	▼	ns	ns	ns*	ns	S*	S
BG01912	*Pxb*	In gene (exon)	89A1-A2	ns	▼	ns*	ns	ns	ns	▲*	ns	S	S
BG01990	*CG43340*	61 bp at 5´ side	43E6-E7	ns	▼	ns	▼*	ns	▼	ns	ns	ns	S*
BG02042	*easily shocked* (*eas*)	In gene (5´UTR)	14B7	ns	ns	ns	ns*	ns	ns	▲	ns	ns	S
BG02088	*CG15309*	In gene (intron)	9B4	ns	ns	ns	▼	ns	▼	ns	ns	ns	S*
BG02102	*mir-313, 312, 311, 310 / mir-2498, 991, 992*	69, 208, 369, 489 bp at 5’ side / 380, 871, 985 bp at 3’ side	57A6	ns	▼	ns	ns	ns	▼	ns	ns	ns	S
BG02106	*CG31145*	In gene (5´UTR)	95A4-A7	ns	ns	ns	ns	ns	▼	▲	ns	ns	S*
BG02157	*CG42268*	46 bp at 5´ side	67C6	ns*	ns	ns	ns	ns	ns	▲	ns	ns*	S

(Continued)

Table 1 continued

BG02159	*Death-associated protein kinase related* (*Drak*)	In gene (5´UTR)	10C1-C5	ns*	▼	ns*	▼	ns	▼	ns	ns	ns	S
BG02239	*CG11550*	9536 bp at 5' side	100D1	ns*	▼*	ns*	▼*	ns*	ns*	▲*	ns	S	S
BG02462	*CG34460*	1317 bp at 3´ side	53D11	ns*	▼*	▼*	▼*	▼	▼	ns	ns	S*	S*
BG02563	*Capricious* (*caps*)	In gene (5´UTR)	70A3-A4	ns	ns	ns	ns	ns	ns	▲	ns	ns*	ns
BG02690	*CG14478*	In gene (intron)	54B16	ns*	ns	ns	▼*	ns	▼	ns	ns*	S*	S*
BG02747	*rutabaga* (*rut*)	In gene (5´UTR)	12F4-F5	ns	▼	▼	▼	▼	ns	ns	ns	S	S
BG02823	*scylla* (*scyl)*	546 bp at 5' side	68B4-68C1	ns	▼	ns	▼	ns	▼	▲	ns	S	S
BG02830	*Lipid storage droplet-2* (*Lsd-2*)	379 bp at 5' side	13A8-A9	ns	▼*	ns*	▼*	▼*	▼*	ns*	ns*	S*	S*

The candidate gene, the site of the mutation and its morphological effect are shown. ns: not significant. For body size related traits, ▼ and ▲ represent significant decrements and increments with respect to the control respectively ($p<0.05$). For wing shape, S represents significant differences with respect to the control ($p<P_{Bonferroni}=0.0012$). * Significant at 25°C [12, 13].
