# Supplementary material for: Gene-by-Temperature Interactions and Candidate Plasticity Genes for Morphological Traits in Drosophila melanogaster
Source: PLoS One. 2013 Jul 30;8(7):e70851. doi: 10.1371/journal.pone.0070851 (PMC3728209; doi:10.1371/journal.pone.0070851)
Supplement: Table S2 — Principal results of genetic correlation analyses between body size related traits at 17°C and 25°C. Correlation coefficients corresponding to the analyses within each sex and between sexes for each variable are shown. (PDF) [file pone.0070851.s006.pdf]

Table S2. Principal results of genetic correlation analyses between body size related traits at 17°C and 25ºC.

	Face Width	Head Width	Thorax Length	Wing Size
17ºC				
Face Width	*0.70****	**0.74*****	**0.72*****	**0.73*****
Head Width	0.81***	*0.73****	**0.86*****	**0.91*****
Thorax Length	0.81***	0.80***	*0.73****	**0.86*****
Wing Size	0.78***	0.88***	0.83***	*0.77****
25ºC				
Face Width	*0.69****	**0.32***§	**0.42****	**0.06** ns
Head Width	0.59***	*0.37**§	**0.84*****	**0.76*****
Thorax Length	0.26 ns	0.75***	*0.14* ns	**0.60*****
Wing Size	0.33*§	0.75***	0.73***	*0.72****

A correlation analysis was performed between each pair of variables within males (bold-faced *r* values above the diagonal), within females (underlined *r* values below the diagonal) and between sexes for each variable (italized *r* values on the diagonal). ns: not significant; *p<0.05; **p<0.01; ***p<0.001. § Not significant after Bonferroni correction for multiple tests (P_B=0.0125).
