# Supplementary material for: Gene-by-Temperature Interactions and Candidate Plasticity Genes for Morphological Traits in Drosophila melanogaster
Source: PLoS One. 2013 Jul 30;8(7):e70851. doi: 10.1371/journal.pone.0070851 (PMC3728209; doi:10.1371/journal.pone.0070851)
Supplement: Table S3 — Principal results of ANOVAs for morphological traits in each temperature and sex separately and analyses of GEI. The F values and the genetic variance components derived from the ANOVAs are shown. Also, the correlation coefficients and the components explaining the interaction between temperatures are given. (PDF) [file pone.0070851.s007.pdf]

Table S3. Principal results of ANOVAs for morphological traits in each temperature and sex separately and analyses of GEI.

Trait	*F* Line$_{25°C}$	*F* Line$_{17°C}$	r_{GxE}	V_{GxE} (S-R)
Females				
Face Width	4.16 (18)	6.43 (25)	0.19	1.09-98.91
Head Width	5.68 (24)	7.42 (28)	0.22	0.08-99.92
Thorax Length	6.44 (27)	8.36 (31)	0.12	0.43-99.57
Wing Size	6.61 (28)	7.92 (29)	0.40	0.04-99.96
Wing Shape	2.83 (11)	5.43 (20)	0.30	1.28-98.72
Males				
Face Width	7.32 (30)	4.48 (17)	0.38	16.95-83.05
Head Width	6.78 (28)	12.22 (40)	0.33	0.14-99.86
Thorax Length	4.96 (21)	10.10 (35)	0.49	0.08-99.92
Wing Size	10.01 (38)	11.12 (37)	0.30	0.12-99.88
Wing Shape	4.30 (19)	4.40 (16)	0.64	8.96-91.04

The *F* value ($p<0.000001$ in all cases) and the percentage of total phenotypic variation explained by the line factor (between parentheses) are shown. Also, the correlation coefficient (r_{GxE}) between different environments (17°C and 25°C) and the percentage of variance corresponding to GEI (V_{GxE}) explained by a change in scale (S) and a change in ranking order (R) are shown.
