# Supplementary material for: Gene-by-Temperature Interactions and Candidate Plasticity Genes for Morphological Traits in Drosophila melanogaster
Source: PLoS One. 2013 Jul 30;8(7):e70851. doi: 10.1371/journal.pone.0070851 (PMC3728209; doi:10.1371/journal.pone.0070851)
Supplement: Table S4 — Principal results of the ANOVAs performed to study the change of the phenotypic effect of the P -element insertion with thermal change in each line, sex and trait separately. The unsigned difference between the means of the transformed values at 17°C and 25°C for each morphological trait are given. (PDF) [file pone.0070851.s008.pdf]

Table S4. Principal results of the ANOVAs performed to study the change of the phenotypic effect of the *P*-element insertion with thermal change in each line, sex and trait separately.

	Face Width		Head Width		Thorax Length		Wing Size		Wing Shape	
**Line**	♂	♀	♂	♀	♂	♀	♂	♀	♂	♀
BG00369	0.021	0.083*	0.014	**0.062****	0.011	0.092**	0.046*	0.039*	0.305*	**0.486****
BG00373	0.058*	**0.123****	0.053*	**0.097****	**0.073****	**0.129****	0.022	**0.088****	0.240*	**0.795****
BG00386	0.03	**0.160****	0.036*	0.048*	0.011	0.047*	0.055**	0.011	0.315*	0.200*
BG00524	0.064*	0.018	0.016	0.008	0.012	0.029*	0.037*	0.012	**0.480****	0.469**
BG00737	**0.145****	**0.134****	0.032*	0.060**	0.071**	0.046*	0.013	0.004	0.108	0.269*
BG00930	0.057*	0.093*	**0.054****	**0.067****	0.041*	0.073**	0.024*	0.043*	0.081	0.380**
BG00992	0.035	0.037	0.017	0.012	0.041*	0.049**	0.033*	0.009	0.135	0.129
BG01011	0.007	0.046	0.039*	0.014	0.064**	0.069*	0.011	0.019	0.325*	0.314*
BG01014	0.038*	0.047	0.004	0.047*	0.033*	0.045*	0.044**	0.01	0.261*	0.283*
BG01028	0.025	0.027	0.039*	0.012	0.023	0.052**	**0.070****	0.012	0.085	**0.547****
BG01081	0.050*	0.001	0.007	0.007	0.032*	0.035*	0.048**	0.035*	0.224*	0.385**
BG01214	0.001	0.103**	0.024*	0.032*	0.033*	0.058**	0.015	0.014	0.06	0.131*
BG01218	0.064	0.084**	**0.044****	0.032*	**0.082****	0.067**	0.017	0.037*	0.079	0.237*
BG01290	0.064*	0.068**	0.042**	**0.069****	**0.078****	**0.108****	0.009	**0.053****	0.153*	0.184*
BG01339	0.075*	0.075**	**0.063****	0.034*	0.034	0.042*	0.008	0.008	0.276**	**0.512****
BG01354	0.029	**0.152****	0.031*	0.058**	0.048**	0.081**	0.000	**0.055****	0.094	0.261**
BG01488	0.02	0.047	0.019	0.03	0.055*	0.073**	0.009	0.044*	0.236	0.218*
BG01548	0.063**	0.013	0.044*	0.009	0.011	0.035*	**0.071****	0.01	0.164	0.341**
BG01573	0.005	0.053	0.052*	0.037	0.082*	**0.099****	0.023	0.023	0.251**	0.333**
BG01672	0.061*	**0.138****	0.004	0.031	0.043*	0.006	0.045**	0.011	0.354**	0.213*
BG01683	0.047*	0.051	0.015	0.003	0.005	0.038	0.062**	0.035	0.239*	0.336*
BG01716	0.014	0.023	0.003	0.014	0.028*	0.029*	0.042**	0.008	0.012	0.055
BG01726	0.03	0.027	0.024*	0.005	0.048**	0.049**	0.007	0.002	0.063	0.366**
BG01735	0.056*	0.090**	0.007	0.018*	0.021	0.028	0.054**	0.007	0.149	0.198*
BG01780	**0.092****	0.108**	0.021	0.001	0.053*	0.014	**0.087****	0.036*	0.240*	0.399**
BG01822	0.012	0.040*	0.019	0.012	0.039*	0.035*	0.004	0.001	0.229**	0.308**
BG01902	0.017	0.044*	0.051*	0.01	0.036	0.03	**0.075****	0.012	**0.561****	0.361**
BG01912	0.063*	0.088**	0.038**	0.039*	0.035*	0.036*	0.013	0.006	0.297*	0.359**
BG01990	0.015	0.055*	0.009	0.005	0.031	0.056**	0.021	0.013	0.256*	0.349**
BG02042	0.005	0.036	0.01	0.039*	0.018	0.014	0.040*	0.043*	0.043	0.082
BG02088	0.091*	0.079*	0.008	0.045	0.033	0.021	0.045*	0.011	**0.514****	0.275*
BG02102	0.022	0.066*	0.019	0.001	0.043*	0.096**	0.002	0.051*	0.128	0.310*
BG02106	0.01	0.044*	0.014	0.006	0.041*	0.048*	0.021*	0.007	0.267*	0.420*
BG02157	0.07	0.049*	0.009	0.007	0.001	0.03	0.067**	0.009	0.335*	0.068
BG02159	**0.082****	0.116**	**0.051****	**0.086****	0.060**	**0.100****	0.007	**0.035****	0.184*	0.279**
BG02239	**0.075****	0.018	0.042**	0.031*	**0.078****	**0.102****	**0.077****	0.018	0.085	0.101
BG02462	0.048*	0.064*	0.018	0.041*	0.028	0.076**	0.032*	0.02	0.203*	0.324**
BG02563	0.000	0.015	0.02	0.008	0.021	0.011	0.031*	0.028	0.370*	0.263*
BG02690	**0.138****	0.039	0.011	0.060*	0.030*	0.017	0.030*	**0.072****	**0.391****	**0.615****
BG02747	0.063*	0.045	**0.096****	0.044*	**0.072****	0.021	0.039*	0.021	**0.390****	0.469**
BG02823	0.042*	0.044*	0.009	0.034*	0.009	0.052**	0.035**	0.017	0.186**	0.283*
BG02830	0.028	0.016	0.042*	0.022	0.003	0.002	0.068**	0.049*	0.216**	0.368**

Values represent the unsigned differences between the means of the transformed values at 17ºC and 25ºC. Bold-faced values correspond to the five largest significant values in each case. *$p<0.05$; **$p<P_B=0.0012$.
